# Simultaneous quantification of alpha-aminoadipic semialdehyde, piperideine-6-carboxylate, pipecolic acid and alpha-aminoadipic acid in pyridoxine-dependent epilepsy

**DOI:** 10.1038/s41598-019-47882-2

**Published:** 2019-08-06

**Authors:** Jiao Xue, Junjuan Wang, Pan Gong, Minhang Wu, Wenshuang Yang, Shiju Jiang, Ye Wu, Yuwu Jiang, Yuehua Zhang, Tatiana Yuzyuk, Hong Li, Zhixian Yang

**Affiliations:** 1 Department of Pediatrics, Peking University First Hospital, Beijing, China; 2 Zhejiang Biosan Biochemical Technologies Co., Ltd, Zhejiang, China; 3 Department of Clinical Laboratory, Peking University First Hospital, Beijing, China; 4 Department of Pathology, University of Utah, Salt Lake City, UT USA; 5 ARUP Laboratories, ARUP Institute for Clinical and Experimental Pathology, Salt Lake City, UT USA; 6 Department of Human Genetics, Emory University, School of Medicine, America, Atlanta, USA

**Keywords:** Diagnostic markers, Epilepsy, Epilepsy

## Abstract

The measurements of lysine metabolites provide valuable information for the rapid diagnosis of pyridoxine-dependent epilepsy (PDE). Here, we aimed to develop a sensitive method to simultaneously quantify multiple lysine metabolites in PDE, including α-aminoadipic semialdehyde (a-AASA), piperideine-6-carboxylate (P6C), pipecolic acid (PA) and α-aminoadipic acid (α-AAA) in plasma, serum, dried blood spots (DBS), urine and dried urine spots (DUS). Fifteen patients with molecularly confirmed PDE were detected using liquid chromatography-mass spectrometry (LC-MS/MS) method. Compared to the control groups, the concentrations of a-AASA, P6C and the sum of a-AASA and P6C (AASA-P6C) in all types of samples from PDE patients were markedly elevated. The PA and a-AAA concentrations ranges overlapped partially between PDE patients and control groups. The concentrations of all the analytes in plasma and serum, as well as in urine and DUS were highly correlated. Our study provided more options for the diverse sample collection in the biochemical tests according to practical requirements. With treatment modality of newly triple therapy investigated, biomarker study might play important role not only on diagnosis but also on treatment monitoring and fine tuning the diet. The persistently elevated analytes with good correlation between plasma and DBS, as well as urine and DUS made neonatal screening using DBS and DUS possible.

## Introduction

Pyridoxine-dependent epilepsy (PDE; OMIM 266100), a rare autosomal recessive disorder, is caused by mutations in the gene coding for aldehyde dehydrogenase 7 A1 (*ALDH7A1*), also known as antiquitin^1^. In mammals, antiquitin involves in lysine catabolism where it metabolizes α-aminoadipic semialdehyde (α-AASA) to α-aminoadipic acid (α-AAA)^2^. With antiquitin dysfunction, metabolic intermediates of lysine catabolism pathway including α-AASA, piperideine-6-carboxylate (P6C) and pipecolic acid (PA) accumulate, which are markedly elevated in urine, plasma and cerebrospinal fluid, and can be used as diagnostic markers of PDE. Moreover, the potential toxicity of these accumulating compounds might directly cause cellular injury and further contribute to the neurodevelopmental impairments in PDE^3^. The combination of a lysine-restricted diet with pyridoxine and arginine supplements, known as triple therapy, decreased accumulation of PDE biomarkers and improved cognitive and motor development in majority of reported patients^4,5^. Plasma levels of the sum of AASA and P6C (AASA-P6C) and PA directly correlated with plasma lysine^5^. Therefore, the measurements of a-AASA, P6C and PA provide valuable information for the rapid diagnosis and monitoring the treatment of patients with PDE, as well as prognosis prediction. Recently, Wempe *et al*.^6^ reported a novel biomarker 6-oxo-pipecolate (6-oxo-PIP) for PDE that was stable at room temperature, while the sensitivity and specificity of it still need further studies.

The measurement of a-AASA, P6C and PA was still limited to few laboratories world widely. Several studies on the determination of plasma and urinary a-AASA and/or P6C by liquid chromatography-mass spectrometry (LC-MS/MS) method, and determination of PA by gas chromatography-mass spectrometer (GC-MS) or LC-MS/MS were previously published^1,7–9^. Moreover, detection of a-AASA and P6C in dried blood spots (DBS) from newborn patients with PDE using LC-MS/MS or a novel method HILIC-ESI-MS were reported^10,11^. However, these analytes in different types of samples (such as plasma, urine and DBS), or even different analytes in the same sample, were usually need to be measured by different methods with various specimen preparation and turned around time. In a 2016 publication, Pena *et al*.^12^ described a protocol that used LC-MS/MS to simultaneously detect the PA, P6C as well as saccharopine, glutamic acid and pyridoxal-5′-phosphate in mouse plasma samples. Thus far, only one study on simultaneous detection of AASA-P6C and PA in plasma and urine from PDE patients by LC-MS/MS were reported^13^. Here, we modified the method described previously^13^, and made it suitable for the simultaneous quantification of multiple metabolites including a-AASA, P6C, AASA-P6C, PA and α-AAA in plasma, serum and DBS as well as urine and dried urine samples (DUS), which could be clinically used for early diagnosis and monitoring patients with PDE.

## Material and Methods

### Ethics statement

This study was approved by the Biomedical Research Ethical Committee of Peking University First Hospital, and written informed consents were obtained from the legal guardians (parents) of the children. All experiments were performed in accordance with relevant guidelines and regulations. All data were analyzed anonymously.

### Patient and control samples preparation

A total of five types of samples from 15 patients with PDE were collected, including plasma (n = 15), serum (n = 14), DBS (n = 15), urine (n = 15) and DUS (n = 15). All samples were freshly collected before meal and taking pyridoxine. Then, blood and urine were spotted onto filter paper card and allowed to dry at room temperature for about four hours to prepare DBS and DUS. Blood aliquots were also used to prepare plasma and serum samples. No serum sample was collected from patient 14. The clinical features and genotypes of 7 out of the 15 PDE patients (patient 3, 5, 6, 8, 9, 11, 15) have been reported previously^14,15^. All patients were on daily pyridoxine supplements (30–360 mg/d) without specific diet restriction at the time of testing. The psychomotor development was assessed according to clinical judgment and the restandardization of the Adaptive Scale of Infant and Children^16^.

Unaffected controls samples were collected from the patients with genetic generalized epilepsy (excluding PDE genetically), tic disorders or simple upper respiratory infection. Considering that all our PDE patients were older than 1 year at the time of the testing, we selected the children aged 1–13 years old as controls. Only samples with normal findings on routine biochemical tests and/or metabolic screening were included. The control ranges for plasma (n = 28), serum (n = 25), DBS (n = 25), urine (n = 25) and DUS (n = 25) were established. Because the P6C concentration of one control plasma sample was unusually much higher than the others (about 15 times) and there was not enough sample volume for the retest, we did not include it into statistics.

The specimens were then kept on dry ice during shipment and then stored at −80 °C until analysis.

### The determination of the lysine metabolites using LC-MS/MS

#### Reagents

The following reagents were purchased: hydrochloic acid, 3Nin n-butanol (Regis Technologies, Inc.), methanol and acetonitrile (meker, us), formicacid, a-AAA, PA, 5-sulfosalicylic acid dihydrate Amberlyst® 15 dry resin and allysine-ethylene acetal (AEA) (Sigma), d9-PA and d3-a-AAA (CDN Isotopes).

#### AASA-P6C synthesis

Lacking commercially available standards, the AASA-P6C reference material (a mix of a-AASA and P6C) was synthesized from AEA using Amberlyst® 15 bead according to published procedures^1,8,13^. The efficiency of the conversion of AEA to AASA-P6C and residue traces of AEA were confirmed as the published procedure^8^. According to these results, the final concentration of AASA-P6C in reference material was calculated based on the assumption of 100% of synthesis efficiency. Reproducibility of AASA-P6C synthesis was evaluated by the peak areas ratio of a-AASA and P6C for the same amounts of synthesized material from the different batches on three different days. We used 1:3 ratio to approximate a-AASA and P6C concentrations based on assumption that the ionization efficiency of a-AASA and P6C were close as the published procedure^8,13,17^.

#### Control samples and calibration

Six non-zero calibrators were prepared at concentrations of 2–400 umol/L for AASA-P6C, 0.5–100 umol/L for PA and 0.5–100 umol/L for a-AAA in the buffer (2.5% BSA and 0.8% NaCl), normal urine or 30% acetonitrile in water for the use with plasma, DBS or urine, respectively.

Three plasma quality controls (QC) were prepared by spiking normal plasma with AASA-P6C, a-AAA and PA standards at low (5 µmol/L), medium (20 µmol/L) and high (100 umol/L) concentrations. To prepare three DBS QC, normal whole blood was spiked with the same levels of AASA-P6C, a-AAA and PA, and spotted onto filter paper card (Protein Saver™ 903® Card, Whatman Inc, Piscayaway, NJ). After drying at room temperature overnight, DBS were stored in sealed plastic bags. Three QCs were prepared in urine at low (5 mol/L AASA-P6C, a-AAA and PA), medium (20 mol/L AASA-P6C and AAA, 50 mol/L PA) and high (200 mol/L AASA-P6C and a-AAA, 100 mol/L PA) concentrations. The three DUS QCs were prepared as the same concentrations as in urine QCs by soaking filter paper card and drying at room temperature overnight. All types of QCs were stored at −80 °C.

#### Plasma, urine and DBS specimens

An aliquot of 20 μL of urine was mixed with 120 μL of acetonitrile containing 15.0 μM of d3-a-AAA and d9-PA as the internal standards. An aliquot of 50 μL of plasma was mixed with 220 μL of acetonitrile containing 7.5 μM of d3-a-AAA and d9-PA as the internal standard. Three DBS (ID 3 mm) was mixed with 150 μL of 50% methanol containing 10.0 μM of d3-a-AAA and d9-PA as the internal standard. DUS specimens were prepared as the urine after reconstituted by water.

The mixture was vortexed for 2 min, allowed to stand for 3 min, and then centrifuged at 20000 g at 4 °C for 10 min. An aliquot of supernatant was transferred to a clean tube and dried by nitrogen flow in room temperature. The residue was derivatized with 100 μl of 3 N HCl in n-butanol (v/v) at 65 °C and 600 rpm for 30 min dried as described above and reconstituted in 100 μL water/methanol (70:30) containing 0.1% of formic acid.

#### Detection methods

**Figure 1:**
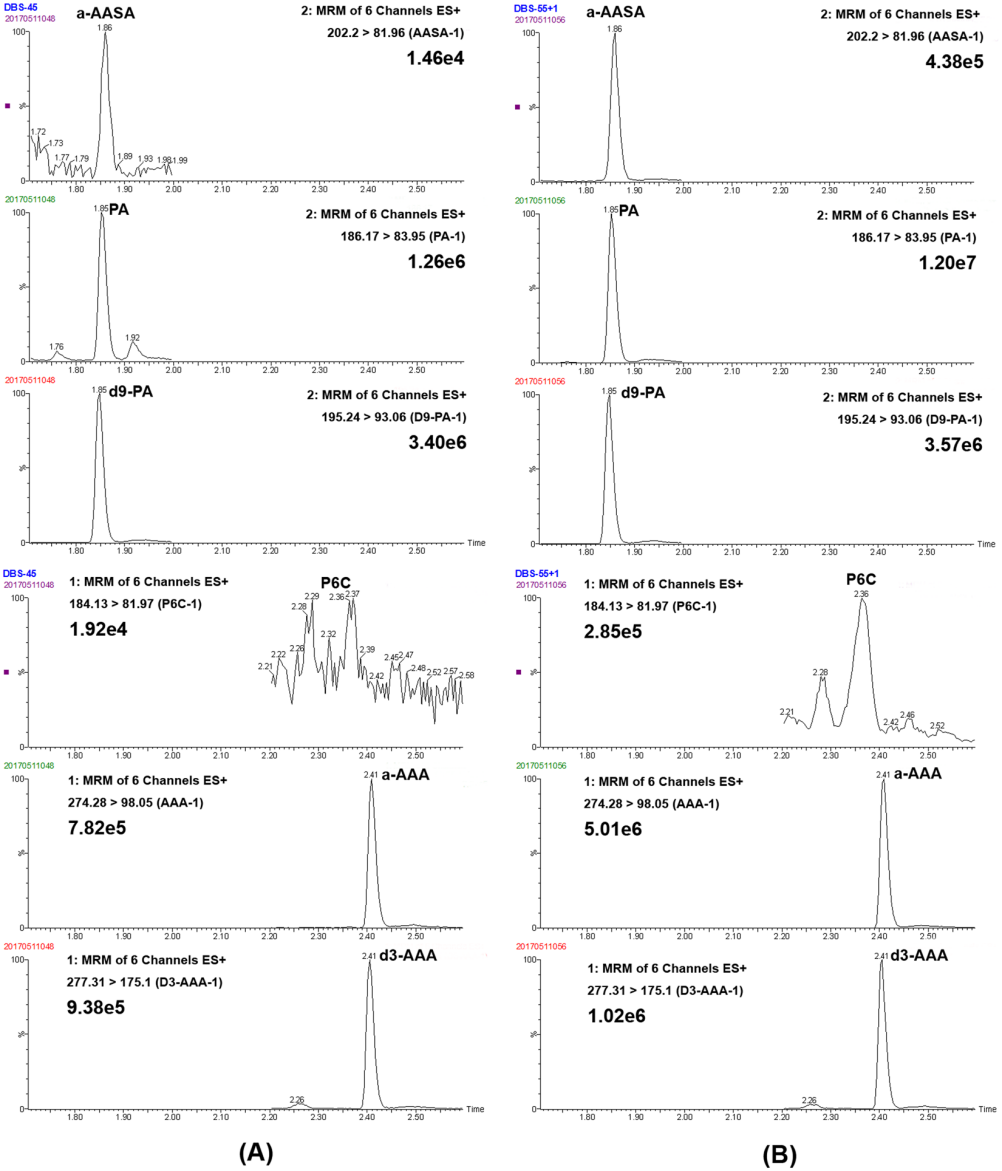
The classic extract ion chromatogram of a-AASA, P6C, PA, a-AAA, d9-PA and d3-AAA in control and PDE patient in DBS samples.

Separation was achieved on an ACQUITY BEH-C18 column (2.1 × 50 mm, 1.7 m) at a temperature of 40 °C using a linear gradient of mobile phase A (0.1% of formic acid in water) and mobile phase B (0.1% of formic acid in methanol) as follows: 0 min, 10% B (0.4 mL/min); 1.5 min, 45% B (0.3 mL/min); 2.5 min, 75% B (0.3 mL/min); 3 min, 95% B (0.3 mL/min); 4–5 min, 10% B (0.3 mL/min). The mass spectrometer was operated in positive ion mode on a Waters Xevo TQD MS/MS with a 1.2 kV capillary voltage. The source and desolvation gas temperature was 150 °C and 550 °C, respectively. The data were acquired in with multiple reaction monitoring (MRM) mode. The cone and collision energy for the detection of a-AASA, P6C, a-AAA, d3-a-AAA, PA and d9-PA were shown in Table S1. The examples of extract ion chromatogram (EIC) of a-AASA, P6C, PA, a-AAA, d9-PA and d3-AAA in control and PDE patient in DBS, plasma and urine samples were shown in Figs 1, S1–2, respectively.

#### LOD/LOQ

The limit of detection (LOD) was determined by analyzing plasma, DBS, and urine lowest control samples by diluting concentrations progressively (n = 6) until a minimum signal-to-noise ratio (S/N) of 3 was achieved. The limit of quantification (LOQ) for all analytes was determined as LOD until a minimum signal-to-noise ratio (S/N) of 10 was achieved. Usually, LOQ is defined as the lowest concentration of analyte, at which the measured concentration is within ±10–30% of the target concentration with the precision (CV) of <10–20%.

#### Precision

Three replicates of each QC level for plasma, DBS and urine were used to calculate the intra-assay precision. The inter-assay precision is evaluated by analyzing the samples over five days.

#### Stability test for plasma, urine, DBS and DUS

Whole blood and urine samples were collected from five healthy adult volunteers in EDTA tubes, and then pooled according to the sample types. QC plasma samples were prepared by spiking normal plasma with the following concentrations of added AASA-P6C: 4.8, 6.4, 16 μmol/L, and separately PA and AAA: 2, 4, 16 μmol/L. Urine QC samples were prepared by spiking normal urine with the following concentrations of added AASA-P6C: 15, 25, 65 μmol/L, and separately PA and AAA: 5, 20, 80 μmol/L. DBS samples were prepared by spiking whole blood with the following concentrations of added AASA-P6C: 4, 12, 24 μmol/L, and separately PA and AAA: 5, 15, 30 μmol/L. DUS samples were prepared by spiking urine matrix with the following concentrations of added AASA-P6C: 4, 20, 60 μmol/L, and separately PA and AAA: 5, 25, 75 μmol/L. DBS and DUS samples were spotted onto filter paper card (Protein Saver™ 903® Card, Whatman Inc, Piscayaway, NJ), allowing to dry at room temperature for about four hours and then stored in sealed plastic bags. The stability of AASA-P6C, a-AAA and PA in plasma, urine, DBS and DUS were tested at room temperature over 7 days, and at 4 °C over 30 days. Two PDE patients’ urine, plasma, DBS and DUS samples stored in −80 °C over 8 months were also tested to get the stability of analytes.

#### Statistical analysis

All statistical analysis were completed using SPSS 16.0. The Shapiro-Wilk test was used to test whether variables were normally distributed. A Student’s t-test (2-tailed) or Mann-Whitney U test was used to test differences in a-AASA, P6C, AASA-P6C, PA and a-AAA concentrations between patients and control group. The association of the metabolites between different samples was analyzed by calculating the correlation coefficient, and *R*^2^ > 0.7 was considered to be a strong correlation. The significance level was set at 0.05 and all tests to assess *P* values were two-sided.

## Results

### Analytical performance of the method

The intra-assay precision of AASA-P6C, a-AAA, and PA in plasma, DBS and urine were within 10%. The inter-assay precision of AASA-P6C, a-AAA, and PA in urine were 1.9–6.2%, 2.6–8.7% and 2.7–5.9% respectively. The inter-assay precision of AASA-P6C, a-AAA, and PA in plasma were 4.1–5.9%, 7.0–9.8% and 4.0–10.3% respectively. The inter-assay precision of AASA-P6C, a-AAA, and PA in DBS were 3.4–5.8%, 2.8–4.2% and 4.4–5.7% respectively. The results for LOQ and LOD were shown in Table S2.

### Stability of plasma, urine, DBS and DUS

**Figure 2:**
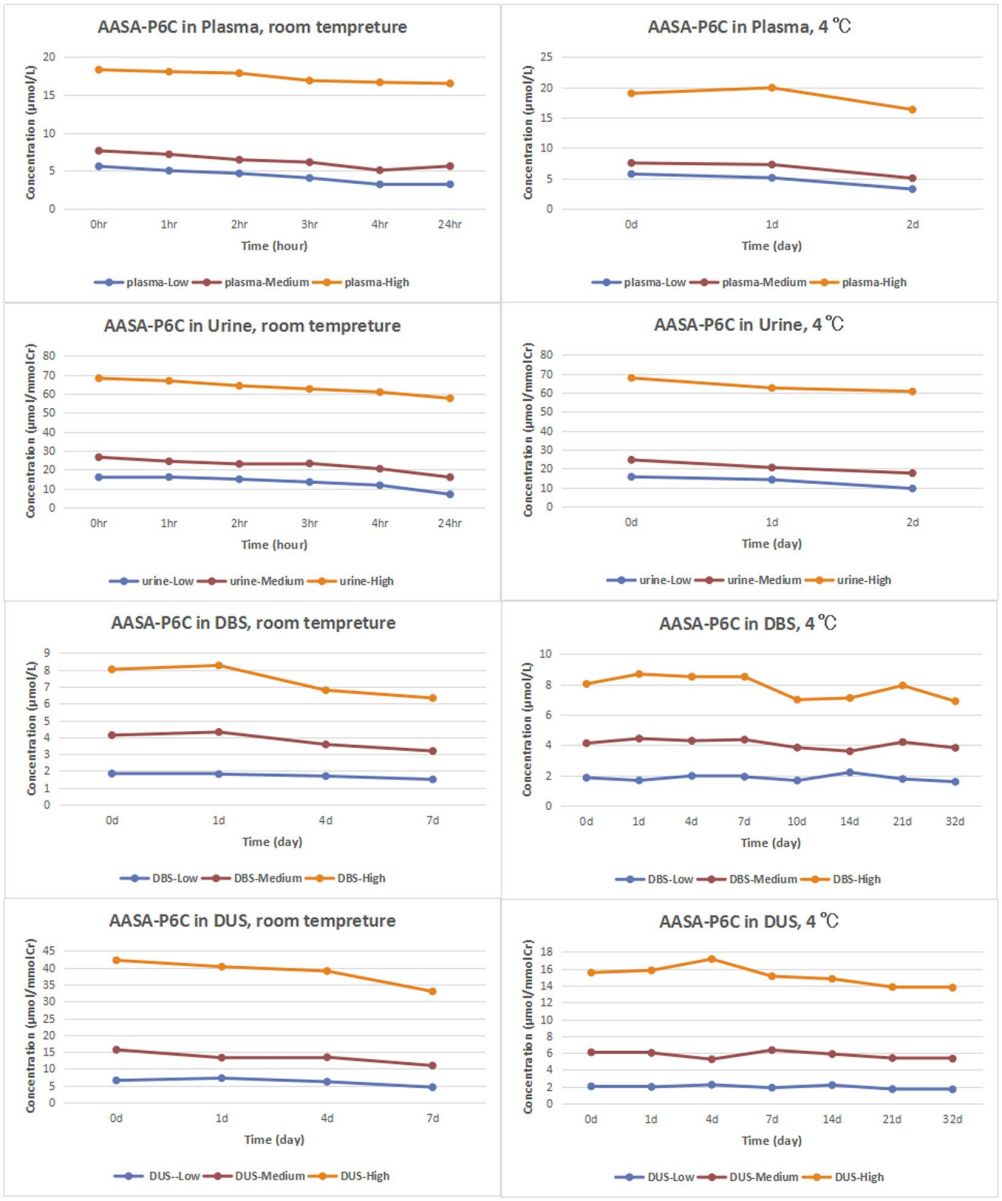
The stability of AASA-P6C in plasma, urine, DBS and DUS at room tempreture and 4 °C, respectively.

As the Fig. 2 shown, when the plasma and urine samples were stored either at room temperature for 24 hours or 4 °C for 2 days, the low, medium and high level of AASA-P6C dropped to below 60%, 70% and 80% of the initial value respectively. When DBS and DUS were stored at room temperature after 7 days, the low, medium and high AASA-P6C concentration were above 70% of the initial value. When DBS and DUS were stored at 4 °C after 32 days, the low, middle and high AASA-P6C concentration were above 85% of the initial value. The concentrations of PA and α-AAA in the plasma, urine, DBS and DUS were above 80% and 85% of the initial value at room temperature and 4 °C respectively. The plasma, urine, DBS and DUS samples of two PDE patients were stored at −80 °C for 8 months, and the concentrations of AASA-P6C, PA and α-AAA in all samples are above 85% of the initial value. In conclusion, DBS and DUS samples were more stable than plasma and urine at room temperature and 4 °C, allowing for more reliable clinical screening.

### General information of PDE patients

A total of 15 PDE patients were included in this study (1.3–8.6 years old) (Table 1). All patients were confirmed the diagnosis through mutation analysis of *ALDH7A1* and being treated with oral pyridoxine at the time of testing, at doses of 30–360 mg/d. Neurodevelopmental evaluation showed 10 out of 15 patients had expressive speech delay, most with mild delay (7/10), two with moderate delay and one with severe delay; Only 2 showed motor delay, all others were normal on motor development.

**Table 1:** Summary of clinical and molecular findings of the fifteen PDE patients.

No./Sex	Seizure onset age	Age at test	Pyridoxine dose	Duration of pyridoxine treatment	*ALDH7A1* mutations (NM_001182.4)	Development		Xue *et al*., 2015^14^	Xue *et al*., 2016^15^
						Language	Motor		
1/F	3m	4y3m	360 mg/d	3y10m	c.1061 A > G (p.Y354C); Deletion of exon 8–13	Moderate	Normal	/	/
2/M	3.5m	8y7m	200 mg/d	4y	c.1553 G > C (p.R518X); c.1061 A > G (p.Y354C)	Normal	Normal	/	/
3/M	23d	3y4m	90 mg/d	3y	c.1279 G > C (p.E427Q); c.1279 G > C (p.E427Q)	Mild	Normal	Mild/1y4m	Mild/1y11m
4/F	6m	4y5m	180 mg/d	4m	c.1547 A > G (p.Y516 C); c.212 C > T (p.P71L)	Normal	Normal	/	/
5/F	2d	3y	150 mg/d	3y	c.1008 + 1 G > A (IVS11 + 1 G > A); c.796 C > T (p.R266X)	Mild	Normal	Mild/1y	Moderate/1y6m
6/M	1d	5y6m	180 mg/d	3y9m	IVS17-1_7delCCACTAG + c.1566_1567delTA; c.871 + 5 G > A (IVS9 + 5 G > A)	Moderate	Normal	Severe/3y5m	Severe/4y
7/F	1y1m	5y4m	180 mg/d	1y4m	c.1279 G > C (p.E427Q); c.986 G > A (p.R329K)	Mild	Normal	/	/
8/F	8d	6y1m	180 mg/d	5y2m	c.965 C > T (p.A322V); c.952 G > C (p.A318P)	Normal	Normal	Mild/4y	Mild/4y7m
9/M	2m	5y8m	180 mg/d	5y1m	c.410 G > A (p.G137E); c.1008 + 1 G > A (IVS11 + 1 G > A)	Normal	Mild	Severe/3y7m	Severe/4y2m
10/F	1d	4y5m	150 mg/d	4y	c.1415 + 1 G > T (IVS15 + 1 G > T); c.871 + 5 G > A (IVS9 + 5 G > A)	Mild	Normal	/	/
11/M	1d	3y10m	120 mg/d	3y	c.1531 G > A (p.D511N); c.1008 + 1 G > A (IVS11 + 1 G > A)	Severe	Normal	Severe/1y9m	Severe/2y3m
12/F	5m	5y3m	240 mg/d	3y5m	c.1061 A > G (p.Y354C); c.1008 + 1 G > A (IVS11 + 1 G > A)	Mild	Normal	/	/
13/M	1m	4y9m	90 mg/d	2m	c.1547 A > G (p.Y516 C); c.1061 A > G (p.Y354C);	Mild	Mild	/	/
14/F	1.5m	1y4m	180 mg/d	11m	c.1547 A > G (p.Y516 C); c.1547 A > G (p.Y516 C)	Normal	Normal	/	/
15/M	8d	2y6m	30 mg/d	2y	c.1547 A > G (p.Y516 C); c.1072 C > T (p.R358X)	Mild	Normal	/	Mild/1y

### Concentrations of PDE biomarkers in PDE patients and unaffected controls

Control samples were collected from the patients with genetic generalized epilepsy (excluding PDE genetically), tic disorders or simple upper respiratory infection (1–13 yrs). The mean and ranges of the five metabolites concentrations in each type of control samples were shown in the Table 2. The a-AASA concentrations in plasma and serum samples were too low to determine here, which were recorded as 0.00 µmol/L.

**Table 2:** The ranges of the metabolites concentrations in PDE patients and control groups.

	Samples	α-AASA	P6C	α-AASA-P6C	PA	α-AAA
**PDE patients/(1.3–8.6y), n = 15**	Plasma (µmol/L)	4.89 (0.59–14.57)	5.99 (2.95–11.58)	10.87 (4.40–24.17)	4.45 (1.49–8.12)	4.05 (2.19–6.69)
	Serum (µmol/L)	3.86 (0.28–11.36)	5.93 (3.25–10.17)	9.79 (3.73–20.65)	4.72 (1.63–8.29)	4.49 (2.55–6.95)
	DBS (µmol/L)	3.80 (1.17–9.49)	0.64 (0.38–1.56)	4.44 (2.30–10.04)	3.38 (1.38–5.81)	5.64 (2.66–9.45)
	Urine (µmol/mmolCr)	31.4 (1.81–94.35)	10.53 (1.41–27.66)	41.95 (3.22–122.00)	0.13 (0.02–0.38)	27.99 (12.25–74.89)
	DUS (µmol/mmolCr)	30.05 (2.07–86.32)	10.44 (2.69–28.55)	40.49 (4.76–114.87)	0.13 (0.01–0.37)	28.54 (10.64–75.47)
**Controls/(1–13y)**	**Samples/mean ages (ranges)**	**α-AASA**	**P6C**	**α-AASA-P6C**	**PA**	**α-AAA**
	Plasma (µmol/L)/n = 28	0.00	0.40 (0.25–0.58)	0.40 (0.25–0.58)	1.50 (0.38–7.61)	2.22 (1.04–4.72)
	Serum (µmol/L)/n = 25	0.00	0.35 (0.34–0.378)	0.35 (0.34–0.38)	0.91 (0.33–3.52)	2.00 (0.97–4.23)
	DBS (µmol/L)/n = 25	0.02 (0.00–0.08)	0.17 (0.15–0.23)	0.19 (0.15–0.26)	0.81 (0.17–3.37)	1.60 (0.91–2.90)
	Urine (µmol/mmolCr)/n = 25	0.12 (0.00–0.53)	0.43 (0.10–1.10)	0.55 (0.18–1.11)	0.17 (0.00–0.94)	15.92 (2.62–51.12)
	DUS (µmol/mmolCr)/n = 25	0.10 (0.00–0.67)	0.41 (0.10–1.13)	0.51 (0.18–1.13)	0.31 (0.01–1.19)	19.72 (2.51–53.70)

**Figure 3:**
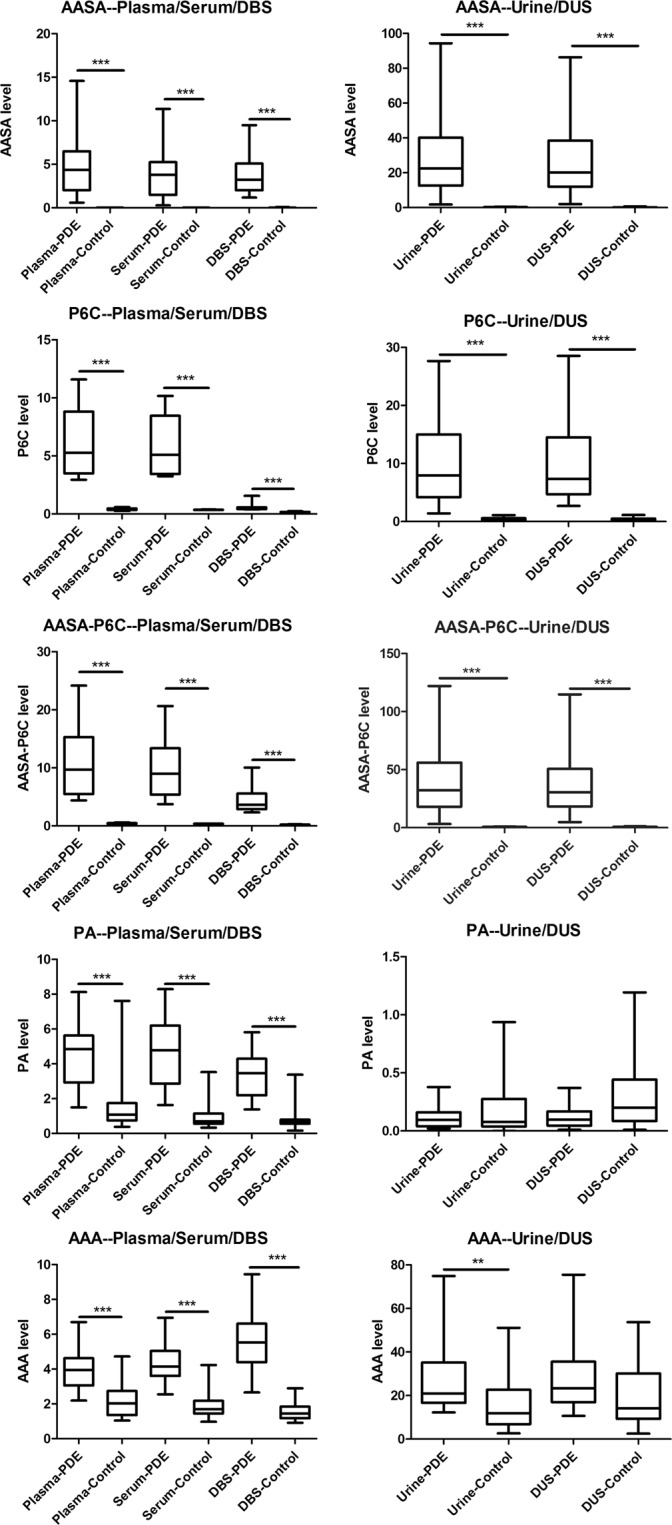
Box-plot of a-AASA, P6C, AASA-P6C, PA and a-AAA in plasma, serum, DBS, urine and DUS from unaffected controls and confirmed PDE patients.

The concentrations of biomarkers varied significantly among different PDE patients, and the detailed data were shown in Supplementary Dataset. Compared to the control groups, the a-AASA, P6C and AASA-P6C in all types of samples were markedly elevated in the PDE patients (*p* < 0.001). In PDE patients, the mean concentration of PA was elevated in plasma, serum and DBS (*p* < 0.001), and mean concentrations of a-AAA was elevated in plasma, serum and DBS (*p* < 0.001), as well as in urine (*p* < 0.05). However, the PA and a-AAA concentration ranges overlapped partially between PDE patients and control groups in all specimens (Fig. 3). And as for every PDE patient, PA and a-AAA could be moderate elevated or within normal range.

### Comparison the metabolites concentrations between different samples in PDE patients

The concentration of a-AASA (*R*^2^ = 0.958, *p* < 0.001), P6C (*R*^2^ = 0.968, *p* < 0.001), AASA-P6C (*R*^2^ = 0.981, *p* < 0.001), PA (*R*^2^ = 0.919, *p* < 0.001) and a-AAA (*R*^2^ = 0.940, *p* < 0.001) was positively correlated between plasma and serum. Similarly, the concentration of the a-AASA (*R*^2^ = 0.987, *p* < 0.001), P6C (*R*^2^ = 0.961, *p* < 0.001), AASA-P6C (*R*^2^ = 0.988, *p* < 0.001), PA (*R*^2^ = 0.929, *p* < 0.001) and a-AAA (*R*^2^ = 0.987, *p* < 0.001) in urine and DUS was positively correlated also. The PA (*R*^2^ = 0.958, *p* < 0.001), a-AASA (*R*^2^ = 0.733, *p* < 0.001) and AASA-P6C (*R*^2^ = 0.826, *p* < 0.001) concentrations were highly correlated between DBS and plasma samples, but no obvious correlation was found in P6C (*R*^2^ = 0.039, *p* = 0.241) and a-AAA (*R*^2^ = 0.419, *p* = 0.005) concentrations (Fig. S3). Compared the metabolites concentrations in DBS and plasma, the P6C, AASA-P6C and PA levels were higher in plasma (*p* < 0.001), and a-AAA level was higher in DBS (*p* < 0.001).

## Discussion

Early recognition and diagnosis of PDE were highly desirable as prompt treatment could maximize benefits^18,19^. The availability of biomarker testing would facilitate early diagnosis of this treatable condition. This might be also potentially applicable to newborn screening. Currently, most researches on biochemical detection were focused on one or several analytes, and restricted to the limited number of PDE patients^8,10,17^. Here, we simultaneously detected a-AASA, P6C, AASA-P6C, PA and a-AAA from five different types of samples, including plasma, serum, DBS, urine and DUS, freshly collected from 15 patients with PDE, which was the most comprehensive study in a relative larger cohort at present. The reference range of each metabolite in PDE patients and control groups were determined, providing possible medical reference ranges for the biochemical screening. The successful detection of the biomarkers in DBS and DUS might promote the establishment of neonatal screening of PDE.

The elevated concentration ranges of a-AASA, P6C and AASA-P6C in plasma and urine were basically in agreement with previously published in PDE patients, which confirmed the reliability of our test^1,7,8,13,20^. In our study, though the mean concentration of PA was elevated in blood specimens in PDE patients, the PA concentration ranges partially overlapped between PDE patients and control groups in all the types of samples. Previous reports suggested that PA levels might be more responsive to pyridoxine treatment and could be normalized after many months to years successful treatment with pyridoxine or with age^8,18,21^. Moreover, it could not be ruled out that in some of our cases, PA levels were normal from the onset as reported previously^22^. In addition, elevated concentrations of PA were also encountered in other conditions, such as generalized peroxisomal dysfunction, hyperlysinemia, defects of proline metabolism, chronic liver dysfunction, or even in patients without any apparent cause, giving it a low specificity^19,23^.

The current research of biochemical detection in PDE patients mainly concentrated on plasma and urine a-AASA, P6C and PA, seldom on the a-AAA, the direct downstream metabolite of a-AASA. At 2003, Baxter reviewed that the a-AAA level was normal in patients with pyridoxine-dependent seizures without genetic diagnosis^24^. Recently, Crowther *et al*.^25^ demonstrated that α-AAA was decreased in fibroblasts from PDE patients compared to controls, and similarly, Coughlin *et al*.^26^ reported that α-AAA production was significantly decreased and correlateed well with α-AASA dehydrogenase activity in an E.coli based expression system with human *ALDH7A1* mutation cloned into. Our study is the first to detect α-AAA levels in samples of PDE patients. Different from those reported previously, in our testing, the a-AAA concentration could be within normal range or even elevated in both the blood and urine specimens for each PDE patient. Based on the limited research at present, we could not judge whether this difference was related to the fact that more inevitable influencing factors existed in human samples than *in vitro* cell culture. The possible explanations for our results were as follows: The degradation of a-AAA is catalyzed by a pyridoxal-5′-phosphate (PLP) dependent transaminase (a-AAA transaminase)^24,27^. In PDE patients, the chemical condensation of the accumulating P6C and PLP lead to a secondary deficiency of PLP, which hampers the degradation of a-AAA to a-ketoadipic acid. Meanwhile, the antiquitin dysfunction reduce the synthesis of a-AAA from a-AASA. It might be the combination effects of these two aspects led to the final result together, which was influenced by different dosage of pyridoxine supplementation and different α-AASA dehydrogenase activity caused by various *ALDH7A1* mutations. In addition, the wide range of a-AAA concentrations in both PDE patients and control groups indicated that it might be susceptible to other factors such as dietary lysine intake and sampling time. However, due to lacking uniform baseline of pyridoxine dosage and lysine restrictions, the explanations above were needed to be further confirmed in the future. Our results showed that a-AAA was not specific enough to be a biomarker of PDE. However, considering the better stability of a-AAA and PA, we suggested that a-AAA and PA could be simultaneously quantified with a-AASA and P6C as an auxiliary index. The individual only with high a-AAA and/or PA level should receive a further detection to avoid missed diagnosis caused by degradation of other biomarkers, though that rarely happens.

The concentrations of a-AASA, P6C, AASA-P6C, PA and a-AAA in plasma, serum, DBS as well as in urine and DUS, varied considerably in our patients. The difference between patients could be several times or even up to dozens of times. This remarkably wide range of metabolites levels had also been reported previously^1,7^. We had no clear explanation for this wide range. It might reflect different levels of a-AASA dehydrogenase residual activity caused by different *ALDH7A1* mutations or dietary protein (L-lysine) intake. Few investigations of the relationship between metabolites concentrations and either pyridoxine dose or neurodevelopmental phenotype in PDE patients have been reported. Sadilkova *et al*.^8^ reported that in five patients with PDE, the one receiving the highest daily dose of pyridoxine had the lowest α-AASA. However, in our study, patient 5 and patient 12 usually had the highest metabolites levels, who had very different onset age (2 days and 5 months, respectively) and had been receiving relative higher daily dose of pyridoxine (150 mg/d and 240 mg/d respectively) for more than 3 years in both; while the lowest metabolites levels were usually present in patient 3 and patient 13, who had been receiving the lower daily dose of pyridoxine (90 mg/d in both) for 3 years and 2 months respectively. Furthermore, all these four patients had mild language and/or motor development delay, without obvious difference in degree. So, our study indicated that no clear correlations existed between the metabolites levels and age (recommend for uses in patients more than 1 year old), pyridoxine dosage or psychomotor development, as well as and onset age and duration of pyridoxine treatment. Recently, triple therapy on PDE patients showed improved biomarkers and phychomotor development, and the direct correlation between lysine levels and PDE biomarkers had been reported^5^. We had not concurrently evaluated the dietary intake because of no specific diet restriction of our patients at the time of sample collected, and did not clear if various lysine intake attributed to the variation.

As shown in Table 1, most patients had normal motor development but different degrees of language barriers, which was consistent with the literature reports that verbal skills were more impaired than nonverbal skills^18,19,28^. The clinical features of 7 of the 15 patients had been described previously^14,15^. During the 2-year follow-up periods from the first study^14^, the psychomotor development of these patients improved with age, and the gaps with normal children were gradually narrowing. This was encouraging as early initiation of treatment was expected to continuously improve long-term prognosis for PDE patients. However, it was a pity that although triple therapy had been reported to be highly efficient in decreased accumulation of PDE biomarkers and improved development in the majority of PDE patients^4,5^, it had been not yet available in China at present and none of our patients were started on it.

Plasma and urine were the most commonly used specimens for biomarkers determination in PDE patients. There was significant positive correlation between the metabolites concentrations in plasma and serum. When testing, one could choose plasma or serum, depending on the actual situation at that time, for example, the type of samples the other upcoming tests required. The significant positive correlation between the analytes levels in urine and DUS provided more options for choosing urine specimen. Our results here showed the lysine metabolites were more stable in DBS and DUS, than in other types of samples. Additionally, DBS and DUS were easier to be prepared with a small amount of blood and urine, and were ideal alternative samples compared to the others, particularly for newborn. Therefore, the successfully simultaneously detection of the biomarkers in DBS and DUS here suggested possibility of neonatal screening for PDE. This feasibility of newborn screening for PDE had ever been suggested by Jung *et al*.^10^ and Mathew *et al*.^11^, after implementing the detection of a-AASA and P6C in neonatal DBS using LC-MS/MS or a novel method HILIC-ESI-MS respectively. Recently, Wempe *et al*.^6^ reported a novel biomarker 6-oxo-PIP that might be used in newborn screening of PDE. While further studies were needed to establish the sensitivity and specificity of 6-oxo-PIP for PDE. Our data showed that among the metabolites with specificity in PDE (a-AASA, P6C and AASA-P6C), the correlation between plasma and DBS was significantly positive for a-AASA and AASA-P6C, but not for P6C. The level of P6C in DBS was much lower than that in plasma, though the concentration was also high enough to recognize PDE. This indicated that P6C might not be so stable in DBS. Considering the difficulty in precise quantification of a-AASA or P6C respectively due to the spontaneous equilibrium between them, it seemed that AASA-P6C might be the most reliable indicator in screening. However, limited to that all the specimens here were post-treatment, larger difference of the metabolites concentrations was expected between naive patient and control, which require further pilot study before applying to newborn screening. In addition, for the advantages of DBS and DUS in transportation and preservation, no doubt that they were the best choices when the samples need to be transported to other laboratories for testing, or be stored for future use.

In conclusion, a modified method was used to simultaneously quantify the a-AASA, P6C, AASA-P6C, PA and a-AAA in different samples including plasma, serum, DBS, urine and DUS. No definite correlations was found between the metabolites levels and age, pyridoxine dosage or psychomotor development in PDE patients. The reference ranges of the metabolites concentration in each sample were determined for PDE patients and control groups respectively, which provided more options for the diverse sample collection in the biochemical tests according to practical requirements. With treatment modality of newly triple therapy investigated, biomarker study might play important roles not only on diagnosis but also on treatment monitoring and fine toning the diet. Reliable biomarker study also helped the interpretation of variants with unknown significance when genetic test is not definitive. The persistently elevated analytes with good correlation between plasma and DBS, as well as urine and DUS made neonatal screening using DBS and DUS possible.

However, we acknowledged that there were several limitations in our study. First, healthy control samples should be investigated to establish a reference range, and individuals with more wide age ranges should be included into the control group. Second, more PDE patients samples should be studied for validation, especially the samples prior to pyridoxine treatment. And patients and controls under 1 year old should be included to be further analyzed. Third, other factors, such as genotypes, should also be considered and analyzed in the future. Forth, series follow up measurement might provide the information if other factors affected the level of biomarkers, such as dietary intake.

## Supplementary information

Table S1-S2, Fig.S1-S3

The detailed data of a-AASA, P6C, AASA-P6C, PA and a-AAA concentrations in plasma, serum, DBS, urine and DUS.
