# Supplementary material for: Simultaneous quantification of alpha-aminoadipic semialdehyde, piperideine-6-carboxylate, pipecolic acid and alpha-aminoadipic acid in pyridoxine-dependent epilepsy
Source: Sci Rep. 2019 Aug 6;9:11371. doi: 10.1038/s41598-019-47882-2 (PMC6684619; doi:10.1038/s41598-019-47882-2)
Supplement: Supplementary file 1 — Table S1-S2, Fig.S1-S3 [file 41598_2019_47882_MOESM1_ESM.pdf]

Title page

Title: Simultaneous quantification of alpha-aminoadipic semialdehyde, piperideine-6-carboxylate, pipecolic acid and alpha-aminoadipic acid in pyridoxine-dependent epilepsy

Running head: Simultaneous quantification of PDE biomarkers

Authors: Jiao Xue[1], Junjuan Wang[2], Pan Gong[1], Minhang Wu[2], Wenshuang Yang[3], Shiju Jiang[3], Ye Wu[1], Yuwu Jiang[1], Yuehua Zhang[1], Tatiana Yuzyuk[4,5], Hong Li[6], Zhixian Yang[1*]

Affiliation for all authors:

[1]Department of Pediatrics, Peking University First Hospital, Beijing, China.

[2]Zhejiang Biosan Biochemical Technologies Co., Ltd, Zhejiang, China.

[3]Department of Clinical Laboratory, Peking University First Hospital, Beijing, China.

[4]Department of Pathology, University of Utah, Salt Lake City, UT, USA.

[5]ARUP Laboratories, ARUP Institute for Clinical and Experimental Pathology, Salt Lake City, UT, USA.

[6]Department of Human Genetics, Emory University, School of Medicine, America.

Table S1. The cone and collision energy for the detection of a-AASA, P6C, a-AAA, d3-a-AAA，PA and d9-PA.

Name	Q1(m/z)	Q3(m/z)	Cone/V	CE/V	RT/min
**a-AASA**	202.2	81.9	25	25	1.86
**P6C**	184.1	81.9	35	18	2.37
**a-AAA**	274.3	98.1	30	22	2.41
**d3-a-AAA**	277.3	101.1	30	22	2.38
**d9-PA**	195.2	93.1	30	18	1.87
**PA**	186.2	84.1	35	28	1.85

Table S2. The limit of detection (LOD) and limit of quantification (LOQ) for all analytes.

Metabolites (μmol/L)	urine（LOD）	Plasma (LOD)	DBS (LOD)	Urine (LOQ)	Plasma (LOQ)	DBS (LOQ)
**α-AASA**	0.063	0.072	0.020	0.265	0.137	0.090
**P6C**	0.103	0.148	0.046	0.394	0.481	0.189
**PA**	0.026	0.037	0.021	0.103	0.128	0.081
**α-AAA**	0.021	0.032	0.023	0.098	0.116	0.083

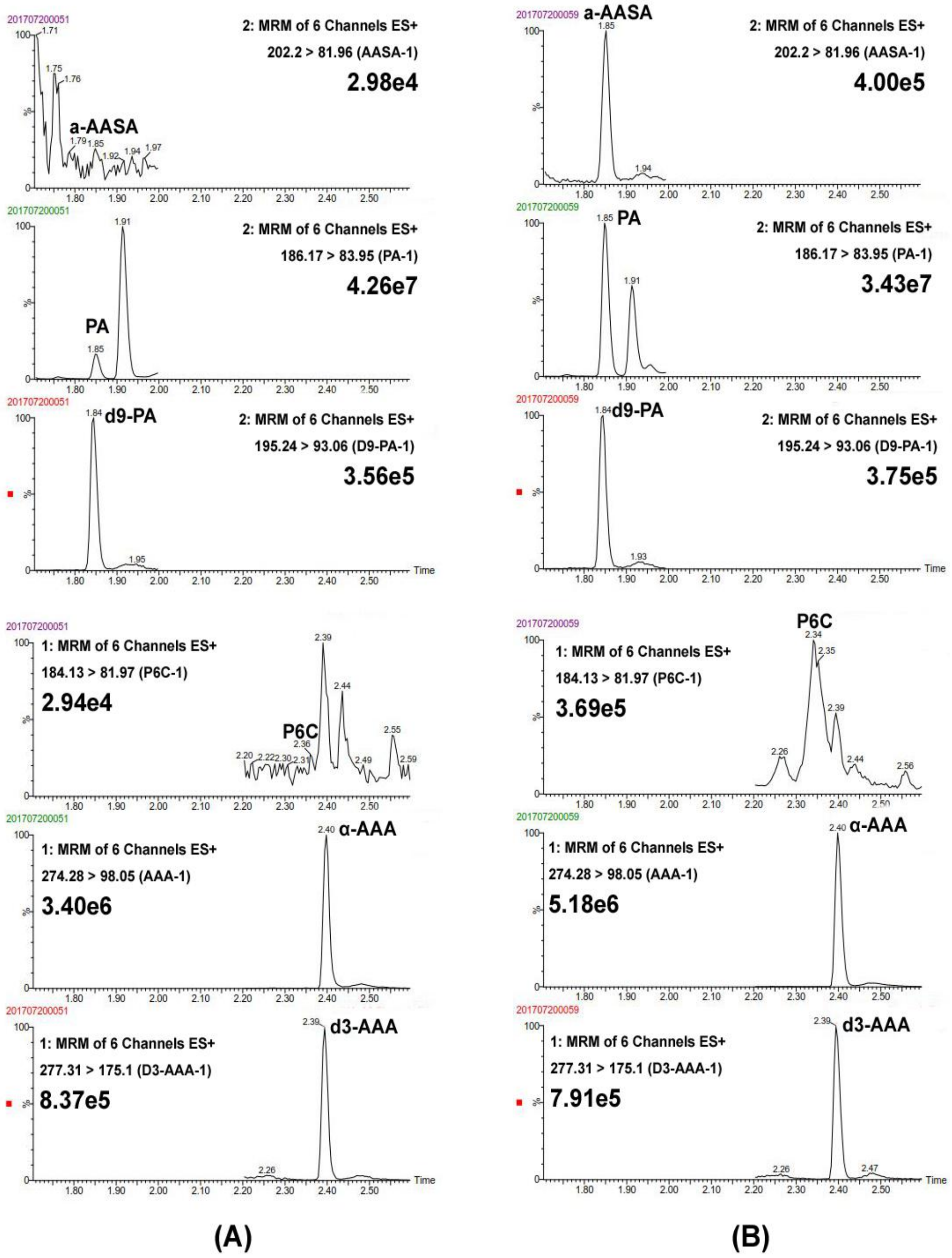

Fig. S1. The classic extract ion chromatogram of a-AASA, P6C, PA, a-AAA, d9-PA and d3-AAA in control and PDE patient in plasma samples.

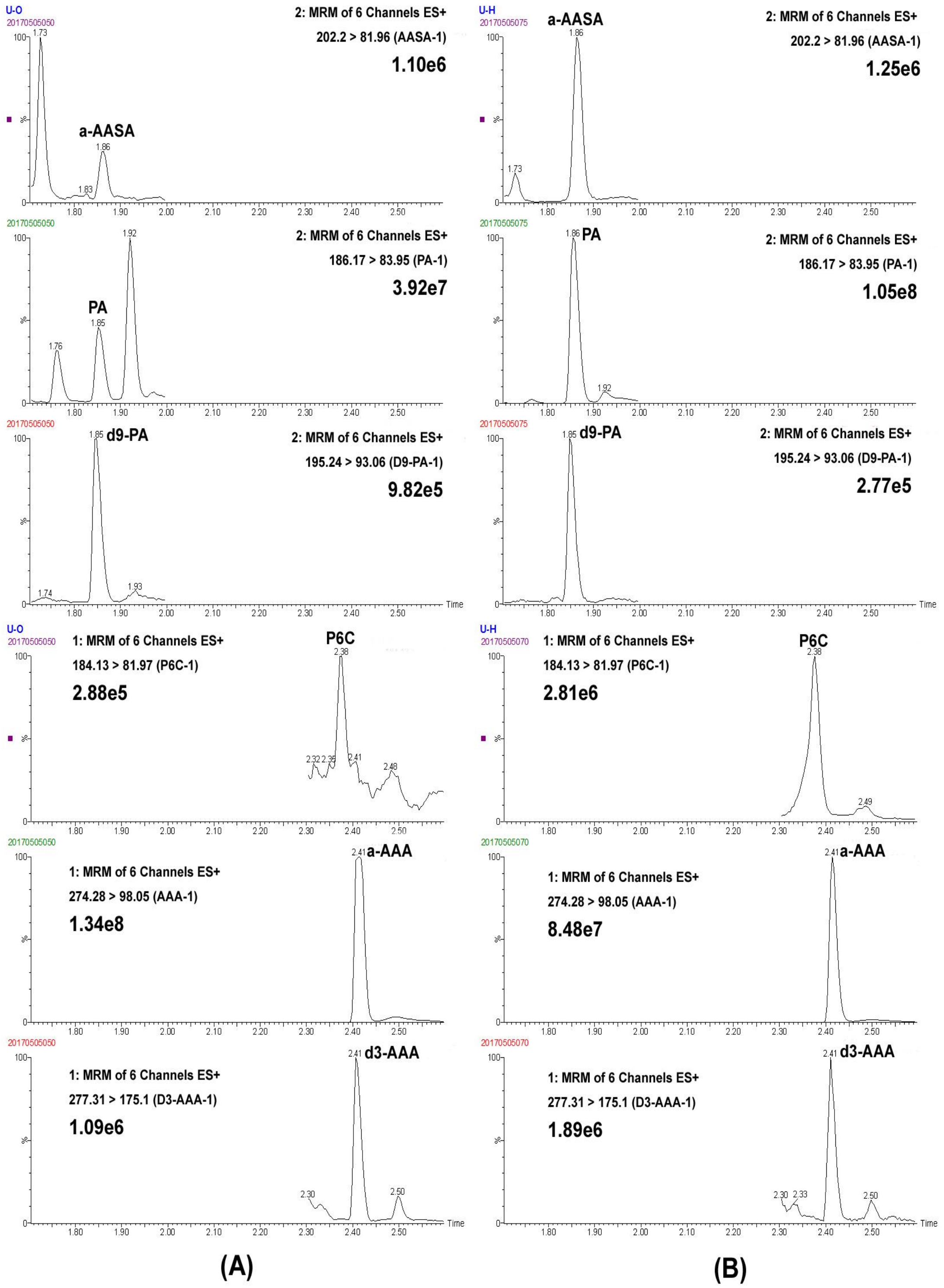

Fig. S2. The classic extract ion chromatogram of a-AASA, P6C, PA, a-AAA, d9-PA and d3-AAA in control and PDE patient in urine samples.

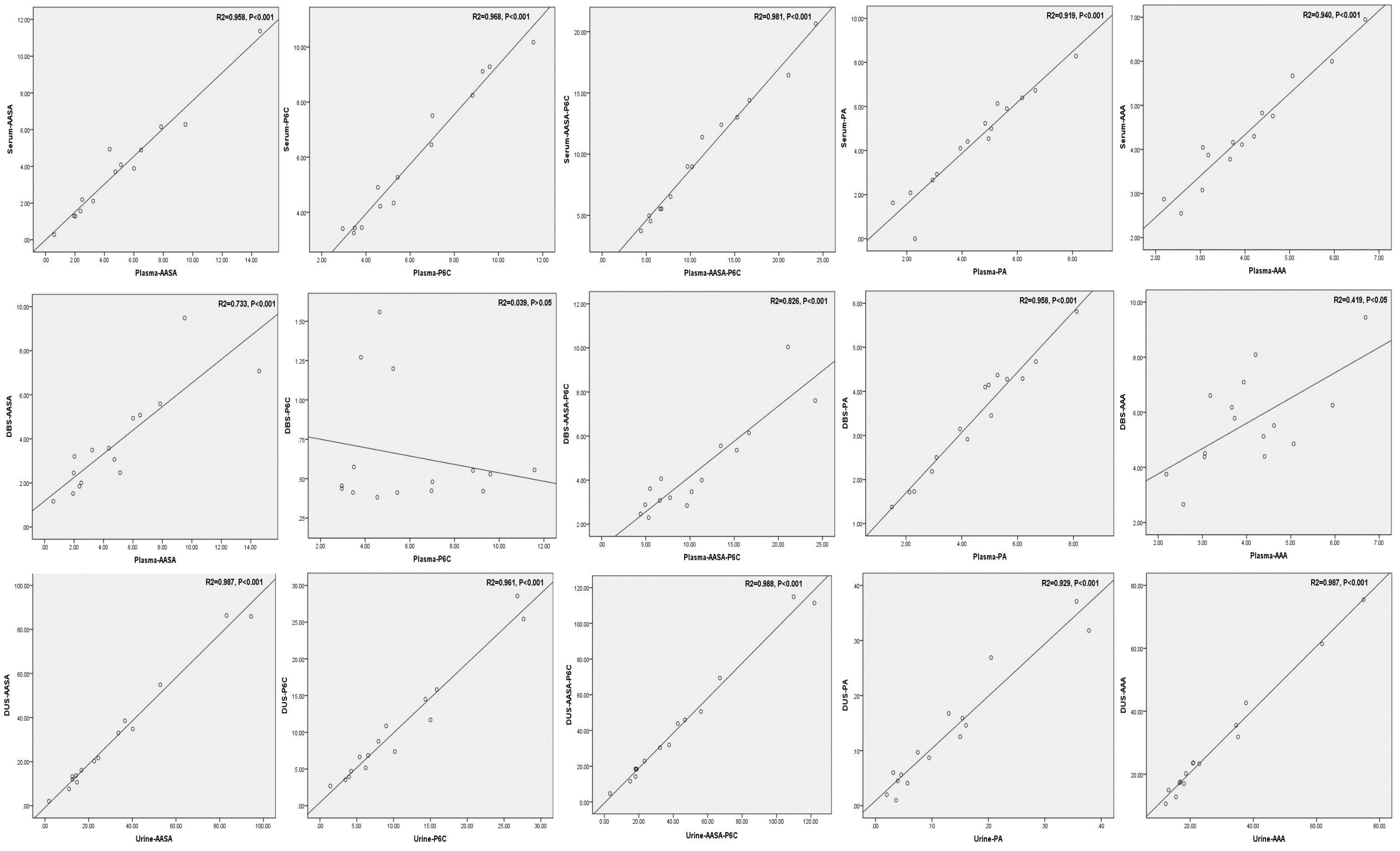

Fig. S3. Correlations of the metabolites concentrations between plasma and serum, plasma and DBS, and urine and DUS.
